# Subpopulations with frequent healthcare barriers have increased risk of sexually transmitted infections and dropping out from HIV preexposure prophylaxis care

**DOI:** 10.1097/QAD.0000000000004224

**Published:** 2025-07-01

**Authors:** Eline S. Wijstma, Vita W. Jongen, Anders Boyd, Henry J.C. De Vries, Maarten F. Schim Van Der Loeff, Maria Prins, Elske Hoornenborg

**Affiliations:** a Department of Infectious Diseases, Public Health Service Amsterdam; b Amsterdam Institute for Immunology & Infectious Diseases (AII); c Stichting HIV Monitoring; d Amsterdam UMC location University of Amsterdam, Department of Infectious Diseases; e Amsterdam Public Health Research Institute (APH); f Amsterdam UMC location University of Amsterdam, Department of Dermatology; g Amsterdam UMC location University of Amsterdam, Department of Internal Medicine, Amsterdam, The Netherlands.

**Keywords:** HIV, preexposure prophylaxis, retention in care, sexually transmitted infections

## Abstract

**Objective:**

Certain subpopulations (i.e., <25 years, transgender, sex worker, uninsured or migrant) were prioritized for inclusion in the Dutch preexposure prophylaxis (PrEP) pilot. We compared incidence of sexually transmitted infections (STI) during and drop-out from HIV PrEP care between prioritized and nonprioritized subpopulations.

**Design:**

Retrospective longitudinal study using routinely collected data at the Centre for Sexual Health Amsterdam, 2019–2024.

**Methods:**

We modelled incidence rates (IR) for any STI while on PrEP using Poisson regression, adjusting for testing frequency and calendar time. We modelled the probability of early loss-to-follow-up (LTFU) (i.e., no PrEP follow-up visit within 12 months of enrolment) using logistic regression, adjusting for sexual behaviour. We modelled the probability of later LTFU (i.e., no PrEP visit within 12 months of a prior PrEP follow-up visit) using competing risk regression, adjusting for sexual behaviour. We added subpopulations as indicator variables to all models to compare endpoints between groups.

**Results:**

Of 4781 individuals included (median age 32 years, IQR = 26–40), 50.2% (*n* = 2402) belonged to prioritized subpopulations. The IR of any STI was 101.6/100 person-years. STI IR were higher among those belonging to prioritized groups (except for the transgender group). 494 individuals had early LTFU, which was associated with age <25 years, reporting sex work, and being a migrant. Later LTFU occurred 933 times and was associated with age <25 years, being transgender, and reporting sex work.

**Conclusion:**

People belonging to prioritized subpopulations had more STIs and were more often LTFU from PrEP care. Targeted interventions to support PrEP retention and prevent STIs are needed.

## Introduction

Oral preexposure prophylaxis (PrEP) with tenofovir and emtricitabine (TDF/FTC) is highly effective in preventing HIV acquisition when taken daily or around the time of sex [1,2]. From 1 July 2019, the Netherlands implemented a 5-year national PrEP pilot program (NPP) to provide subsidized oral PrEP and related care to men who have sex with men (MSM), transgender or gender diverse persons (TGD) and other people with an increased risk for HIV acquisition [3,4]. In the NPP, PrEP consultations were free-of-charge and PrEP was available at a subsidized price of €7.50 for 30 tablets.

The NPP had a maximum capacity of 8500 individuals, which were divided over Centres for Sexual Health based on the estimated number of PrEP-eligible people per region. The Centre for Sexual Health Amsterdam (CSHA) had capacity for 2900 individuals. PrEP care is also provided by general practitioners and private care clinics, but potential users may experience barriers to accessing PrEP care at these points. PrEP care outside the NPP is more expensive for the user (i.e., 30 PrEP tablets cost €17–€65 out-of-pocket and HIV/STI tests are only covered by insurance when healthcare expenditure has surpassed the deductible excess), and potential users may be unable to find a provider willing to prescribe PrEP or face stigma when requesting PrEP [5]. Since these barriers could disproportionately affect PrEP access for certain subpopulations, notably people under the age of 25 years, TGD persons, sex workers, people without health insurance, and migrants from low or middle-income countries (LMIC) or other countries in the global south [6,7], the CSHA prioritized these subpopulations for NPP enrolment.

In a study using NPP enrolment data, we found that individuals from prioritized subpopulations had less prior experience with PrEP, had similar STI prevalence, and accounted for more newly diagnosed HIV infections, compared to nonprioritized subpopulations [8]. These observations highlighted the need to improve access to PrEP care for these subpopulations. Continued engagement in PrEP care for as long as individuals need PrEP is also required to achieve prevention-effectiveness [9]. However, a systematic literature review indicated that some subpopulations, such as young MSM and transgender women, discontinue PrEP earlier [10].

This study aimed to assess the extent to which prioritized and nonprioritized subpopulations enrolled in the NPP in Amsterdam benefitted from PrEP for HIV prevention, by assessing and comparing their STI and HIV incidence while using PrEP. We also examined PrEP their program drop-out and repeat enrolment.

## Methods

### Study design, setting, and population

We conducted a retrospective longitudinal study using routinely collected data from the CSHA. The CSHA is a publicly funded sexual health facility with several locations, including a large central STI test-and-treat location open to any key population (i.e., all MSM, TGD persons, and heterosexual men and women younger than 25), the Amsterdam Centre for Sex Workers, and the Amsterdam Trans Clinic. From 1 July 2019 until 1 August 2024, the CSHA provided subsidized PrEP care as part of the NPP [3].

We included individuals who enrolled in the NPP at the CSHA between 1 July 2019 and 1 November 2023. We excluded individuals who were diagnosed with HIV (if not included as an outcome), individuals who did not commence PrEP for other reasons, and individuals who enrolled in a randomized controlled trial on alternative PrEP care modalities [10].

### Procedures

Enrolment procedures have been described previously [8]. In brief, key populations were made aware of the NPP at CSH facilities and online (i.e., websites of the Public Health Service of Amsterdam, nongovernmental organizations, and community organizations). Interested individuals could submit an online request to enrol in the NPP, or be linked to the NPP when visiting the CSH Amsterdam for other sexual healthcare. Among those interested and PrEP-eligible, individuals from prioritized subpopulations were invited for PrEP intake before those from nonprioritized subpopulations. If enrolled, individuals could choose to use oral PrEP daily or around the time of sex (i.e., 2–1–1 regimen). Users could switch between PrEP regimens at any time, at their own discretion. As part of standard of care, PrEP monitoring visits occurred every three months. After July 2022, the frequency of PrEP monitoring visits could be reduced to every 4–6 months based on shared decision-making between the client and care provider [4]. PrEP monitoring visits included screening for HIV and bacterial STI, a questionnaire on sexual behaviour, and counselling on sexual health and wellbeing. STI and HIV testing in-between PrEP monitoring visits was possible at no additional cost. At each visit, participants could declare PrEP discontinuation or that they would continue PrEP care elsewhere.

For this study, we extracted routinely-collected pseudonymized data on sociodemographic characteristics, sexual behaviour, and STI and HIV diagnoses from electronic health records (EHR). We extracted data from the first PrEP intake visit and all other visits to the CSHA thereafter until 1 February 2024 (i.e., date of data extraction). Informed consent to use pseudonymized surveillance data from EHRs for monitoring and research was obtained through an opt-out procedure. Data are secured in accordance with European privacy legislation. According to national regulations, ethics review is not required for retrospective studies using routinely collected data [11].

### Study variables

Variables relating to priority criteria were: age (<25/≥25 years), gender (TGD/cisgender), sex work in the prior 6 months (yes/no), having no Dutch health insurance (yes/no), and migration background (born in LMIC or other country in the global south [12,13]/born in high-income country (HIC) in the global north; hereafter referred to as ‘born in LMIC’ and ‘born in HIC’). These variables were based on enrolment data and included as time-fixed variables. When information on a given priority criterion was missing, we assumed that the individual did not meet the criterion. We additionally categorized individuals by the number of priority criteria (i.e., 0/1/2/≥3).

Sexual behaviour variables included number of sex partners, any condomless anal sex (CAS) with a casual partner (yes/no), and chemsex (yes/no), all referring to the 6 months before a visit. Casual partners were individuals with whom the client had sex with once or occasionally. We defined chemsex as use of mephedrone, gamma-hydroxybutyrate (GHB), gamma-butyrolactone (GBL), or crystal methamphetamine during or around sex [14]. Sexual behaviour variables were time-updated.

We assessed the number of diagnoses of chlamydia, gonorrhoea, infectious syphilis (stage 1, 2 or recent latent syphilis) and HIV. We defined any bacterial STI as any chlamydia, gonorrhoea or infectious syphilis diagnosis. Concurrent infections of different bacterial STIs were counted separately, while those of the same bacterium at different anatomical sites were counted as one. We defined any anal STI as any anorectal chlamydia or anorectal gonorrhoea diagnosis, whereby concurrent infections of anorectal chlamydia and gonorrhoea were counted separately.

We distinguished four mutually exclusive categories of NPP discontinuations: discontinuation due to HIV diagnosis; ‘formal NPP discontinuation’, defined as a visit whereby an individual declared to discontinue PrEP or continue PrEP care elsewhere; ‘early LTFU’, defined as having no PrEP visit for at least 12 months after first-time NPP enrolment; and ‘later LTFU’, defined as having no PrEP visit for at least 12 months after having attended ≥1 PrEP follow-up visit (i.e., a PrEP visit after first-time or repeat NPP enrolment).

We defined ‘repeat enrolment’ as NPP enrolment after NPP discontinuation.

### Statistical analysis

To examine the incidence of HIV and STI while on PrEP, we included individuals with ≥1 PrEP follow-up visit at the CSHA. Follow-up time began at NPP enrolment or re-enrolment and continued until NPP discontinuation or 1 February 2024, whichever occurred first. IRs were calculated by dividing the number of incident HIV or STI diagnoses by their respective total PY. When calculating the PY for HIV, we assumed that infection occurred at the midpoint between the last negative and first positive test, and time at risk ended after HIV infection. When calculating the PY for STI, we assumed that infection occurred at the date of the positive test, and time at risk continued after infection. To examine differences in STI IR between prioritized and nonprioritized subpopulations, we modelled the STI IRs using Poisson regression with generalized estimating equations (GEE) to account for repeated observations within subjects. In two separate models, we included the five binary indicator variables for priority criteria or an ordinal variable indicating the number of priority criteria, from which incidence rate ratios (IRR) were estimated along with corresponding 95% confidence intervals (CI). We adjusted IRRs for yearly STI testing frequency, calendar time, number of sexual partners (all modelled as cubic splines with four knots at the 5th, 35th, 65th and 95th percentiles), any CAS with casual partners, and chemsex.

To examine the differences in early LTFU between prioritized and nonprioritized subpopulations, we included individuals who enrolled in the NPP in Amsterdam before 1 February 2023 (i.e., who had the opportunity to be considered early LTFU). We then modelled the probability of early LTFU using logistic regression, and estimated odds ratios (OR) along with corresponding 95% CI to compare the odds of early LTFU across levels of covariates. In two separate models, we included the five binary indicator variables for priority criteria or an ordinal variable indicating the number of priority criteria. We adjusted ORs for the number of sex partners (modelled as cubic splines with four knots at the 5th, 35th, 65th, and 95th percentiles), any CAS with casual partners, and any chemsex.

To examine the differences in later LTFU between prioritized and nonprioritized subpopulations, we included individuals who had ≥1 PrEP follow-up visit at the CSHA before 1 February 2023 (i.e., who had the opportunity to be considered later LTFU). Follow-up time began at NPP enrolment and continued until NPP discontinuation or 1 February 2024, whichever occurred first. We calculated the cumulative hazard of later LTFU for prioritized and nonprioritized subpopulations based on the Nelson-Aalen estimator. We then modelled the hazards of later LTFU using proportional hazards regression, while accounting for the competing risks of formally stopping PrEP, seeking PrEP care elsewhere and incident HIV diagnosis using the Fine and Gray method. In two separate models, we included the five binary variables for priority criteria or an ordinal variable indicating the number of priority criteria, from which sub-hazard ratios (HR) comparing the hazards across levels of variables were estimated along with corresponding 95% CI. Standard errors were adjusted for clustered observations among individuals who enrolled multiple times. We adjusted HRs for enrolment period, the number of sex partners, any CAS with casual partners, and chemsex. For each variable, we assessed whether the proportional hazards assumption was met by testing the interaction between ln(time) and covariable using the Wald test.

To examine the differences in repeat NPP enrolment between prioritized and nonprioritized subpopulations, we included all HIV-negative individuals who discontinued the NPP in Amsterdam. Follow-up began at NPP discontinuation and continued until repeat enrolment or 1 February 2024, whichever occurred first. We calculated the IR of repeat enrolment as the total number of repeat enrolment visits divided by the total PY. We then modelled the rate of repeat enrolment using Poisson regression. In two separate models, we included the five binary variables for priority criteria or an ordinal variable indicating the number of priority criteria, from which IRR comparing the IR across levels of variables were estimated along with corresponding 95%CI. Standard errors were adjusted for clustered observations among individuals who discontinued the NPP multiple times.

We described the number of individuals who returned to the CSHA for HIV/STI testing after their NPP discontinuation and, of those, who were diagnosed with HIV.

Statistical significance was defined as *P* < 0.05. All analyses were conducted using STATA (v17, College Station, TX, USA).

## Results

### Study population

**Fig. 1:**
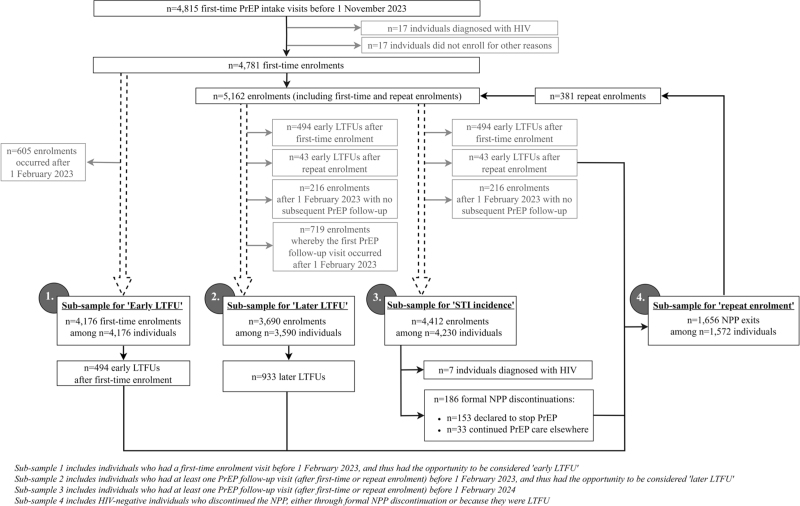
Participant flowchart.

Between 1 July 2019 and 1 November 2023, 4815 individuals had an NPP intake visit at the CSHA (Fig. 1). We excluded 17 individuals who were diagnosed with HIV at this visit and 17 who chose not to commence PrEP for other reasons. Among 4781 individuals included, 2402 (50.2%) belonged to ≥1 prioritized group: 1085 (22.1%) were younger than 25 years, 289 (6.0%) were TGD, 470 (9.8%) reported sex work, 255 (5.3%) had no health insurance, and 1436 (30.0%) were born in LMIC (Table 1). Among the 2402 individuals who belonged to ≥1 prioritized group, the number of priority criteria within individuals was distributed as follows: one, 1712 (71.3%); two, *n* = 400 (16.7%); three, *n* = 178 (7.4%); four, *n* = 100 (4.2%); and five, *n* = 12 (0.5%). There was no multicollinearity between priority variables (tested using variance inflation factor < 5 from a linear regression model). PrEP was most commonly taken as daily regimen (reported at 58.1% of visits, *n* = 19 289), followed by event-driven use (36.6%, *n* = 12 158) or as a combination of both (5.4%, *n* = 1780). Median follow-up time in the NPP was 22 months (IQR 9–34 months; range 0–52 months). Of 1663 NPP discontinuations, seven (0.4%) were due to HIV acquisition; 186 (11.2%) were formal NPP discontinuations (*n* = 153 declared to discontinue PrEP and *n* = 33 continued PrEP care elsewhere), and the remaining 1470 (88.4%) of all discontinuations were due to LTFU (Figure 1).

**Table 1:** Baseline characteristics of individuals enrolled in the national PrEP program in Amsterdam, the Netherlands between 1 July 2019 and 1 November 2023.

	Total (*n* = 4781)	Prioritized (*n* = 2402)	Nonprioritized (*n* = 2379)	
				
	*n* (%)	*n* (%)	*n* (%)	*P*-value^k^
Priority criteria^a^				
Younger than 25 years	1058 (22.1%)	1058 (44.0%)	0 (0.0%)	<0.001
Transgender or gender-diverse	289 (6.0%)	289 (12.0%)	0 (0.0%)	<0.001
Sex work^b^	470 (9.8%)	470 (19.6%)	0 (0.0%)	<0.001
No health insurance	255 (5.3%)	255 (10.6%)	0 (0.0%)	<0.001
Born in LMIC^c^	1436 (30.0%)	1436 (59.8%)	0 (0.0%)	<0.001
Number of priority groups^d^				<0.001
0	2379 (49.8%)	0 (0.0%)	2379 (100.0%)	
1	1712 (35.8%)	1712 (71.3%)	0 (0.0%)	
2	400 (8.4%)	400 (16.7%)	0 (0.0%)	
≥3	290 (6.1%)	290 (12.1%)	0 (0.0%)	
Other sociodemographic characteristics				
Age in years, median (IQR)	32 (26–40)	27 (23–34)	36 (30–47)	<0.001
Region of birth^e^				<0.001
Netherlands	2340 (49.3%)	662 (27.7%)	1678 (71.3%)	
Other Western & Central Europe and North America	952 (20.1%)	291 (12.2%)	661 (28.1%)	
Eastern Europe and Central Asia	110 (2.3%)	110 (4.6%)	0 (0.0%)	
Middle East and North Africa	318 (6.7%)	318 (13.3%)	0 (0.0%)	
Latin America and the Caribbean	649 (13.7%)	649 (27.1%)	0 (0.0%)	
Asia and the Pacific	299 (6.3%)	284 (11.9%)	15 (0.6%)	
Sub-Saharan Africa	77 (1.6%)	77 (3.2%)	0 (0.0%)	
Gender				<0.001
Cigender man	4482 (93.7%)	2106 (87.7%)	2376 (99.9%)	
Cisgender woman	12 (0.3%)	9 (0.4%)	3 (0.1%)	
Transgender man	19 (0.4%)	19 (0.8%)	0 (0.0%)	
Transgender woman	217 (4.5%)	217 (9.0%)	0 (0.0%)	
Nonbinary	36 (0.8%)	36 (1.5%)	0 (0.0%)	
Other	15 (0.3%)	15 (0.6%)	0 (0.0%)	
Education level				<0.001
University/university of applied sciences	3328 (69.6%)	1458 (60.7%)	1870 (78.6%)	
No university/university of applied sciences	1453 (30.4%)	944 (39.3%)	509 (21.4%)	
Sexual behaviour in the preceding 6 months				
Sexual contacts				<0.001
People with penises	4522 (94.6%)	2208 (91.9%)	2314 (97.3%)	
People with penises or vaginas	259 (5.4%)	194 (8.1%)	65 (2.7%)	
Any CAS with casual partner(s) (301 missing)	3237 (72.1%)	1502 (67.2%)	1735 (76.9%)	<0.001
Number of sex partners, quartiles (134 missing)				<0.001
0–3	1219 (26.2%)	626 (26.9%)	593 (25.5%)	
4–7	1136 (24.4%)	525 (22.6%)	611 (26.3%)	
8–15	1153 (24.8%)	517 (22.2%)	636 (27.4%)	
16 or more	1140 (24.5%)	656 (28.2%)	484 (20.8%)	
Chemsex^f^ (45 missing)	1036 (21.9%)	419 (17.7%)	617 (26.1%)	<0.001
Group sex (2912 missing)	714 (38.2%)	409 (36.4%)	305 (40.9%)	0.048
STI diagnosis				
Any STI^g^	724 (15.1%)	366 (15.2%)	358 (15.0%)	0.86
Any anal STI^h^	482 (10.1%)	239 (10.0%)	243 (10.2%)	0.76
Any chlamydia^i^	354 (7.4%)	177 (7.4%)	177 (7.4%)	0.93
Any gonorrhoea^i^	406 (8.5%)	202 (8.4%)	204 (8.6%)	0.84
Infectious syphilis^j^	67 (1.4%)	46 (1.9%)	21 (0.9%)	0.002

CAS, condomless anal sex; IQR, interquartile range; LMIC, low- or middle-income country; NL, Netherlands; PrEP, preexposure prophylaxis; STI, sexually transmitted infection.

a When data on a priority characteristic were missing, we assumed that the individual did not belong to that priority population. Data on priority characteristics were missing for: sex worker (*n* = 315), born in an LMIC (*n* = 40), and no health insurance (*n* = 1668). Of note, health insurance is mandatory in the Netherlands.

b Among 470 sex workers, 278 were cisgender male (59.2%), 166 were transgender female (35.3%), 9 were cisgender female (1.9%), 9 were nonbinary (1.9%), 5 were transgender male, and 3 had another gender identity (0.6%).

c Among 1436 individuals meeting this priority criterion, 1300 (90.5%) were born in countries classified as low- or middle-income according to the 2019 World Bank classification; 136 (9.5%) were born in high-income countries from the Global South.

d Priority groups are not mutually exclusive.

e Categorization based on World Bank Regions (https://datatopics.worldbank.org/sdgatlas/archive/2017/the-world-by-region.html, accessed on 3 December 2024).

f Use of crystal methamphetamine, mephedrone, and/or gammahydroxubutyrate (GHB)/gammabutyrolactone (GBL) around the time of or during sex.

g Chlamydia, gonorrhoea, and/or infectious syphilis (stage 1, 2, or recent latent syphilis).

h Anal chlamydia or anal gonorrhoea.

i Based on urogenital, anorectal, and oropharyngeal samples.

j Syphilis stage 1, stage 2, or recent latent infection.

k *P*-values were based on the Pearson's *χ*^2^ test for categorical variables, and based on the rank-sum test for nonnormally distributed continuous variables.

### Incidence of HIV and sexually transmitted infection during the national preexposure prophylaxis pilot program

4,230 individuals had ≥1 PrEP follow-up visit before 1 February 2024 and were included in analyses on STI and HIV incidence (Fig. 1). Seven individuals were diagnosed with HIV during a total of 8,687 person-years (IR = 0.08/100 person-years, 95% CI = 0.04–0.17). Of those, four belonged to prioritized groups (IR = 0.11/100 person-years, 95% CI = 0.04–0.29) and three did not (IR = 0.06/100 person-years, 95% CI = 0.02–0.19). 8,967 bacterial STIs (*n* = 4679 gonorrhea; *n* = 3627 chlamydia; *n* = 661 infectious syphilis) were diagnosed during 8825 person-years (IR = 101.6/100 PY, 95% CI = 99.4–103.8) (Supplementary Tables 1–2). The incidence of any anal STI was 68.6/100 PY (95% CI = 65.7–71.7).

Incidence of any bacterial STI was higher for individuals with the priority criteria for younger age (aIRR = 1.53, 95% CI = 1.25–1.46), having no health insurance (aIRR = 1.27, 95% CI = 1.08–1.49), and being born in LMIC (aIRR = 1.23, 95% CI = 1.14–1.32) (Table 2, Supplementary Table 3). Incidence of bacterial STI was lower among TGD persons compared to cisgender persons (aIRR = 0.72, 95% CI = 0.60–0.87). The incidence of bacterial STI was higher among individuals who met one, two, or at least three priority criteria compared to individuals who met none (Table 2, Supplementary Table 4). Similar differences were observed for incidence of anal STI and individual STIs (Supplementary Tables 3–4).

**Table 2:** Incidence of bacterial STI^a^ among prioritized and nonprioritized subpopulations enrolled in the national PrEP program in Amsterdam, the Netherlands (1 July 2019–1 February 2024).

	Incidence rates		Incidence rate ratios					
		
			Univariable		Multivariable, model 1^c^		Multivariable, model 2^d^	
					
	IR/100PY [95% CI]	IR/100PY [95% CI]	IRR [95%CI]	p-value^b^	aIRR [95% CI]	p-value^b^	aIRR [95% CI]	p-value^b^
A) Specific priority criteria	Priority criterion met	Priority criterion not met						
Younger than 25 years (vs. ≥25 years)	120.6 [114.9–126.7]	97.9 [95.5–100.3]	1.23 [1.17–1.30]	<0.0001	1.24 [1.14–1.35]	<0.0001	1.53 [1.25–1.46]	<0.0001
TGD (vs. cisgender)	86.5 [76.4–98.2]	102.1 [99.9–104.4]	0.85 [0.74–0.96]	0.0087	0.72 [0.60–0.87]	0.00052	0.75 [0.62–0.90]	0.0026
Sex worker (vs. no sex worker)	137.4 [127.3–148.5]	99.4 [97.2–101.7]	1.38 [1.28–1.49]	<0.0001	1.41 [1.22–1.63]	<0.0001	1.08 [0.93–1.26]	0.30
Uninsured (vs. insured)	140.7 [127.4–155.7]	100.3 [98.1–102.6]	1.40 [1.27–1.55]	<0.0001	1.29 [1.08–1.53]	0.0034	1.27 [1.08–1.49]	0.0030
Born in LMIC (vs. born in HIC)	118.5 [114.0–123.3]	95.3 [92.8–97.8]	1.24 [1.19–1.30]	<0.0001	1.16 [1.08–1.26]	<0.0001	1.23 [1.14–1.32]	<0.0001
B) Number of priority criteria	Number of priority criteria met							
0	90.9 [88.2–93.7]		Ref.		Ref.		Ref.	
1	111.4 [107.5–115.4]		1.23 [1.17–1.28]	<0.0001	1.17 [1.09–1.26]	<0.0001	1.24 [1.16–1.33]	<0.0001
2	137.8 [127.3–149.3]		1.52 [1.40–1.64	<0.0001	1.44 [1.27–1.65]	<0.0001	1.50 [1.32–1.70]	<0.0001
≥3	128.8 [116.3–143.1]		1.42 [1.27–1.57]	<0.0001	1.71 [1.47–1.98]	<0.0001	1.47 [1.24–1.73]	<0.0001

IRR, (adjusted) incidence rate ratio; HIC, high-income country; IR, incidence rate; PY, person-years; CI, confidence interval; LMIC, low- or middle-income country; STI, sexually transmitted infection; TGD, transgender or gender-diverse.

a We defined any bacterial STI as any chlamydia, any gonorrhoea or infectious syphilis, whereby concurrent infections of different bacterial STIs were counted separately with a maximum of three per visit.

b *P*-values were based on the Wald *χ*^2^ test.

c Model 1: adjusted for other priority criteria (only in A), calendar time, and individual yearly STI testing frequency. Calendar time and STI testing frequency were modelled as restricted cubic splines with four knots at the 5th, 35th, 65th, and 95th percentile.

d Model 2: adjusted for other priority criteria (only in A), calendar time, and individual yearly STI testing frequency, number of sexual partners in the preceding six months, any chemsex in the preceding 6 months, and any CAS with a casual partner in the preceding 6 months. Calendar time, STI testing frequency, and number of sex partners were modelled as restricted cubic splines with four knots at the 5th, 35th, 65th, and 95th percentile.

### Early and late loss-to-follow-up during the national preexposure prophylaxis pilot program

**Fig. 2:**
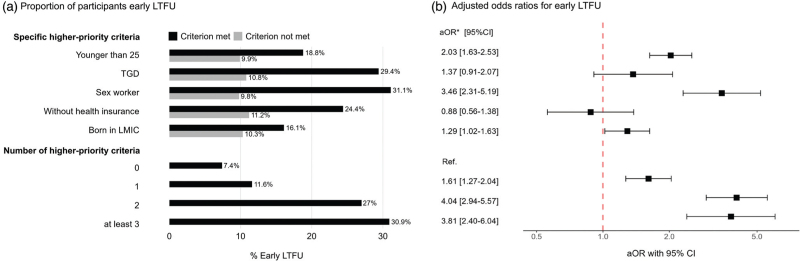
Early LTFU among prioritized and nonprioritized subpopulations who enrolled in the national PrEP program in Amsterdam, the Netherlands (1 July 2019–1 February 2024).

Of the 4176 individuals enrolled before February 2023, 494 (11.8%) had early LTFU (Fig. 1). For each prioritized group, early LTFU occurred more often among individuals who did vs. did not belong to a specific prioritized group (Fig. 2, Supplementary Table 5). After adjusting for the other priority criteria and sexual behaviour, early LTFU was associated with younger age (aOR = 2.03, 95% CI = 1.63–2.53), reporting sex work (aOR = 3.46, 95% CI = 2.31–5.19), and being a born in LMIC (aOR = 1.29, 95% CI = 1.02–1.63). The odds of early LTFU were roughly four times higher among individuals who belonged to two (aOR = 4.04, 95% CI = 2.94–5.57) or at least three (aOR = 3.81, 95% CI = 2.40–6.04) compared to zero prioritized groups.

This figure provides the proportion of individuals who were considered early LTFU (a) and the adjusted odds ratios (aOR) for early LTFU, whereby boxes indicate aORs and lines indicate 95% CIs (b). aOR, adjusted odds ratio; CI, confidence interval; LMIC, low- or middle-income country; LTFU, loss-to-follow-up; *n*, number; n/a, not applicable; NPP, national PrEP pilot; OR, odds ratio; PrEP, preexposure prophylaxis; TGD, transgender or gender-diverse; vs., versus; ^▪^We adjusted ORs for number of sex partners, any condomless anal sex with a casual partner, and any chemsex, all in the past six months. Number of partners was modelled as cubic splines with four knots at the 5th, 35th, 65^th^, and 95th percentile. ORs for the specific priority criteria were also adjusted for the four other priority criteria.

**Fig. 3:**
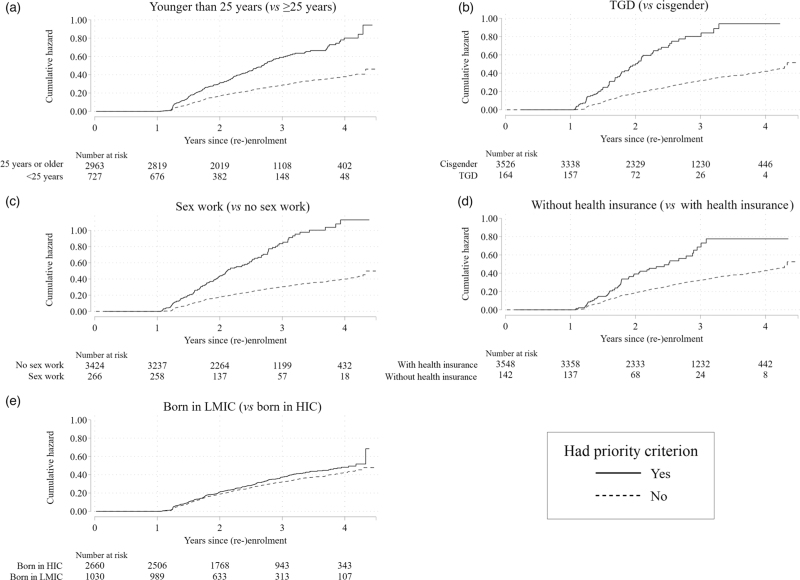
Cumulative hazard function for later LTFU among prioritized and nonprioritized subpopulations who enrolled in the national PrEP program in Amsterdam, the Netherlands (1 July 2019–1 February 2024).

Following 3690 NPP enrolments and repeat enrolments included in the analysis on later LTFU (Fig. 1), there were 933 later LTFUs during 9359 person-years (IR = 0.10 per year, 95% CI = 0.09–0.11). After adjusting for the other priority criteria, enrolment date, and sexual behaviour, later LTFU was associated with meeting the priority criteria for younger age (aHR = 1.69, 95% CI = 1.45–1.97), being TGD (aHR = 1.74, 95% CI = 1.27–2.39), and reporting sex work (aHR = 3.03, 95% CI = 2.31–3.98; Fig. 3, Supplementary Table 6). The subhazards for later LTFU were increasingly higher for individuals who belonged to one (aHR = 1.40, 95% CI = 1.20–1.64), two (aHR = 2.40, 95% CI = 1.91–3.02), or at least three (aHR = 3.72, 95% CI = 2.71–5.12) compared to zero prioritized groups (Supplementary Table 6).

Explanation: the cumulative hazard indicates the total accumulated risk of experiencing LTFU at a specified timepoint *t*. HIC, high-income country; LMIC, low- or middle-income country; LTFU, loss-to-follow-up; PrEP, preexposure prophylaxis; TGD, transgender or gender-diverse; vs., versus. NB: The cumulative hazard function is modelled as the subhazard function, whereby formal NPP exit and HIV diagnosis were competing events to being LTFU.

### Repeat enrolment and HIV after national preexposure prophylaxis pilot program discontinuation

Following 1656 NPP discontinuations among 1572 individuals (Fig. 1), there were 381 repeat enrolments during 2064 person-years (IR = 0.18 per year, 95% CI = 0.17–0.20). The rate of repeat enrolment was higher among individuals from LMIC compared to HIC (aIRR = 1.29, 95% CI = 1.03–1.61), and individuals belonging to at least three vs. zero prioritized groups (aIRR = 1.55, 95% CI = 1.09–2.21) (Supplementary Table 7). During the 2064 person-years after NPP discontinuation, there were 804 individuals who returned to the CSHA for HIV/STI testing but did not re-enrol in the NPP. Of those individuals, two were diagnosed with HIV: one person tested positive at an HIV/STI testing visit, 27 months after being LTFU; one tested positive at a repeat PrEP intake visit, 6 months after being LTFU.

## Discussion

This retrospective longitudinal study in Amsterdam examined key indicators of PrEP care among subpopulations who did or did not meet certain priority criteria for subsidized PrEP care (i.e., age under 25 years, being TGD, reporting in sex work, no healthcare insurance, or being from an LMIC). HIV incidence while on PrEP was low (i.e., 0.08/100 person-years) and comparable between prioritized and nonprioritized groups. STI incidence was higher for each prioritized group, with the exception of TGD persons. All prioritized groups more often had early or later LTFU from PrEP care, but those form LMIC re-enrolled in the program at a higher rate compared to those from HIC. These findings provide quantitative insight into the challenges to retaining prioritized groups in subsidized PrEP programs, and call for targeted interventions to support their PrEP retention.

The vast majority of individuals who discontinued the NPP in Amsterdam were LTFU. LTFU is not an inherently undesirable outcome: in multiple studies from high-income countries, the most common reason for discontinuing oral PrEP was a lower perceived likelihood of acquiring HIV, for example, due to changes in sexual behaviour or engagement with other prevention strategies, such as condoms, U = U, or serosorting [15–22]. However, some studies found that PrEP users may underestimate their risk of acquiring HIV [23], have insufficient knowledge about safe PrEP discontinuation [24], or inconsistently use other HIV prevention strategies after discontinuation [19]. Moreover, several studies, including this one, have reported on individuals who acquired HIV after PrEP discontinuation [22,25–27]. These individuals (similar to most individuals who are LTFU from the NPP) may not have received timely counselling about safe PrEP discontinuation, how and when to restart PrEP if needed, or alternative prevention strategies. As such, tools to help identify when individuals need extra guidance on how to continue PrEP or safely discontinue PrEP are warranted.

Prioritized subpopulations were more often LTFU than nonprioritized subpopulations, both directly after NPP enrolment and later during follow-up. Differences in LTFU remained after adjusting for sexual behaviour, suggesting that differences in susceptibility to HIV did not influence this finding. Prior studies have suggested that some PrEP users have difficulty adhering to quarterly PrEP visits due to work, school, or, more generally, chaotic or stressful life circumstances [15,20,28]. TGD persons in particular may find it burdensome to attend PrEP care in addition to healthcare relating to their medical transition [29], and may discontinue PrEP if they experience covert or overt transphobia while accessing PrEP [30–32]. HIV stigma, which may be particularly high among ethnically minoritized MSM and young MSM, could also hamper PrEP continuation [33]. To support PrEP continuation, programs could provide more personalized PrEP care by offering different frequencies of PrEP monitoring visits, providing opportunities for online and in-person care, and offering short-acting and long-acting PrEP [15,29,34]. Offering PrEP in community-based or peer-led settings could further support PrEP continuation [31,35].

The incidence of bacterial STIs, at 101.6/100PY, was much higher than in other large cohorts of PrEP users [36–38], yet in line with rates observed in previous research in Amsterdam [39]. The incidence rates among prioritized subpopulations were even higher, with the exception of the TGD group. Considering that the presence of STIs is strongly associated with HIV acquisition in MSM who do not use PrEP [40,41], our findings suggest that this cohort of PrEP users, and particularly the prioritized subpopulations of MSM, greatly benefited from PrEP. Moreover, for each prioritized group except those reporting sex work, the association between priority group and STI incidence remained after adjusting for sexual behaviour. This could suggest that the risk of STI acquisition is not only driven by sexual behaviour, but also by varying prevalence of STI between different sexual networks [42]. Taken together, PrEP users in Amsterdam – and especially those belonging to prioritized populations – may benefit from STI prevention strategies. One such strategy could be the use of doxycycline postexposure prophylaxis (doxyPEP), but this is currently not recommended in the Netherlands due to a lack of evidence regarding the long-term effects on antimicrobial resistance and gut microbiome [43].

A quarter of prioritized individuals enrolled in the NPP in Amsterdam had multiple priority criteria. We observed that the effect sizes for early LTFU, later LTFU, and STI increased with the number of higher-priority criteria. This finding aligns with theory of intersectionality, which poses that, as a result of interlocking systems of oppression (such as transphobia, xenophobia, and classism), individuals who belong to multiple marginalized groups often face unique challenges to achieving their health-related goals [44]. Moreover, this observation underscores the importance of considering combinations of characteristics, rather than single characteristics alone, to more accurately assess PrEP-related outcomes across subpopulations.

The NPP closed on 1 August 2024 and PrEP access in the Netherlands has changed considerably since. First, users can no longer obtain PrEP tablets at CSHs, but instead receive a prescription to be presented at a pharmacy. Consequently, STI/HIV testing, counselling, and PrEP tablets can no longer be accessed at one location Second, users pay full price for tablets, which varies between €17 and €65 per 30 tablets depending on the pharmacy, compared to the €7.50 in the NPP [45]. Third, while the NPP allowed anonymous access, pharmacy prescriptions require personal identifiers (including full name and date of birth). This may create barriers, for example, for undocumented persons and sex workers concerned about deportation or prosecution. It is important that PrEP providers take steps to prevent widening disparities in PrEP access.

Our study had several limitations. First, we had no data on the reasons for LTFU, continuation of PrEP elsewhere, or need for PrEP after leaving the NPP. Second, this study only included individuals receiving PrEP via the CSHA. The association between priority characteristics and the outcomes studied may be different in other healthcare settings or other geographic regions [46]. Third, we could not report on HIV incidence after individuals left the NPP due to a lack of data on HIV status after PrEP discontinuation. Finally, the categories used to define priority for subsidized PrEP care are unlikely to fully capture a person's vulnerability to HIV and barriers to PrEP care. Data on socio-economic status, life stability, mental health, health literacy, sexual agency, and outness about one's gender or sexuality would be helpful, but were lacking.

In conclusion, PrEP users who were younger than 25 years, TGD, sex worker, without healthcare insurance, or born in LMIC (i.e., prioritized subpopulations) had a higher incidence of STIs than PrEP users without these characteristics, and were more often LTFU from PrEP care. Nevertheless, HIV incidence within the PrEP program was low. Our findings call for targeted interventions to minimize barriers and optimize facilitators to PrEP continuation for prioritized subpopulations.

## Acknowledgements

### Conflicts of interest

A.B. has received speaker fees from Gilead Sciences, Inc. E.H.'s institute received PrEP medication and unconditional research funding for the AMPrEP study from Gilead Sciences. HJCdV has received speaker fees from AbbVie, served on the advisory committee of Abott Laboratories and was president of the International Society for STD research (ISSTDR). M.P.'s institution has received speaker fees and independent scientific support from Gilead Sciences, Roche, MSD, and Abbvie. MFSvdL has served on Advisory Boards of MSD, Novosanis and Health Council Netherlands, and received funding from GSK for an investigator-initiated study; all payments are made to his institution. E.S.W. and V.W.J. declare no conflicts of interest.

## Supplementary Material

Supplemental Digital Content
