# Supplementary material for: Subpopulations with frequent healthcare barriers have increased risk of sexually transmitted infections and dropping out from HIV preexposure prophylaxis care
Source: AIDS. 2025 Jul 1;39(11):1610–20. doi: 10.1097/QAD.0000000000004224 (PMC12337923; doi:10.1097/QAD.0000000000004224)
Supplement: Supplemental Digital Content [file aids-39-1610-s001.docx]

**Supplementary Tables and Figures for *“Subpopulations with frequent healthcare barriers have increased risk of sexually transmitted infections (STI) and dropping out from HIV pre-exposure prophylaxis (PrEP) care”***

Eline S. WIJSTMA^1,2^, Vita W. JONGEN^1,2,3,4^, Anders BOYD^1,2,3,4^, Henry J.C. DE VRIES^1,2,5,6^, Maarten F. SCHIM VAN DER LOEFF^1,2,5,7^, Maria PRINS^1,2,5,7^, Elske HOORNENBORG^1,2,5,7^

1. Department of Infectious Diseases, Public Health Service Amsterdam, Amsterdam, The Netherlands
2. Amsterdam Institute for Immunology & Infectious Diseases (AII), Amsterdam, the Netherlands
3. Stichting hiv monitoring, Amsterdam, the Netherlands
4. Amsterdam UMC location University of Amsterdam, Department of Infectious Diseases, Amsterdam, the Netherlands
5. Amsterdam Public Health Research Institute (APH), Amsterdam, the Netherlands
6. Amsterdam UMC location University of Amsterdam, Department of Dermatology, Amsterdam, The Netherlands
7. Amsterdam UMC location University of Amsterdam, Department of Internal Medicine, Amsterdam, The Netherlands

**Table of contents**

| Page 2 | **Supplementary Table 1.** Incidence of sexually transmitted infections while on PrEP, among individuals with ≥1 PrEP follow-up visit in the national PrEP program in Amsterdam, the Netherlands (1 July 2019 and 1 February 2024). |
| --- | --- |
| Page 3 | **Supplementary Table 2.** Incidence of sexually transmitted infections while on PrEP, among individuals from prioritized subpopulations with ≥1 PrEP follow-up visit in the national PrEP program in Amsterdam, the Netherlands (1 July 2019 and 1 February 2024). |
| Page 4 | **Supplementary Table 3.** Comparison of the incidence of grouped and specific STIs between prioritized and non-prioritized populations enrolled in the national PrEP program in Amsterdam, the Netherlands (1 July 2019-1 February 2024). |
| Page 6 | **Supplementary Table 4.** Comparison of the incidence of grouped and specific STIs by number of priority criteria, among individuals enrolled in the national PrEP program in Amsterdam, the Netherlands (1 July 2019-1 February 2024). |
| Page 7 | **Supplementary Table 5.** Odds ratios for early LTFU among 4,176 individuals who enrolled in the national PrEP program in Amsterdam, the Netherlands between 1 July 2019 and 1 February 2023. |
| Page 8 | **Supplementary Table 6.** Hazard ratios for later LTFU among 4,230 individuals with ≥1 PrEP follow-up visit before 1 February 2023, in the national PrEP program in Amsterdam, the Netherlands (1 July 2019-1 February 2024). |
| Page 9 | **Supplementary Table 7.** Incidence rate ratios for repeat enrolment among 1,656 individuals who previously exited the national PrEP program in Amsterdam, the Netherlands (1 July 2019-1 February 2024) |

**Supplementary Table 1.** Incidence of sexually transmitted infections while on PrEP, among individuals with ≥1 PrEP follow-up visit in the national PrEP program in Amsterdam, the Netherlands (1 July 2019 and 1 February 2024).

|  | **Total (n=4,230)** | | | | | **No priority criteria  (n=2,198)** | | | | | **One priority criterion (n=1,535)** | | | | | **Two priority criteria (n=300)** | | | | | **At least 3 priority criteria (n=197)** | | | | |
| --- | --- | --- | --- | --- | --- | --- | --- | --- | --- | --- | --- | --- | --- | --- | --- | --- | --- | --- | --- | --- | --- | --- | --- | --- | --- |
|  | No. people with an STI | No. STIs | PY | IR (events/100PY) [95% CI] | | No. people with an STI | No. STIs | PY | IR (events/100PY) [95% CI] | | No.  people  with an  STI | No. STIs | PY | IR (events/100PY) [95% CI] | | No.  people  with an  STI | No. STIs | PY | IR (events/100PY) [95% CI] | | No.  people  with an  STI | No. STIs | PY | IR (events/100PY) [95% CI] | |
| **Any STI**^a^ | 2665 | 8969 | 8825 | 101.6 [99.4-103.8] | | 1404 | 4620 | 5083 | 90.9 [88.2-93.7] | | 954 | 3298 | 2962 | 111.4 [107.5-115.4] | | 184 | 671 | 487 | 137.8 [127.3-149.3] | | 123 | 378 | 293 | 128.8 [116.3-143.1] | |
| **Anal STI**^b^ | 2132 | 6053 | 8825 | 68.6 [65.7-71.7] | | 1118 | 3049 | 5083 | 60.0 [57.9-62.1] | | 759 | 2260 | 2962 | 76.3 [73.2-79.5] | | 155 | 486 | 487 | 99.8 [91.3-109.1] | | 100 | 259 | 293 | 88.3 [78.2-99.7] | |
| **Chlamydia** |  |  |  |  |  |  |  |  |  |  |  |  |  |  |  |  |  |  |  |  |  |  |  |  |  |
| Any chlamydia^c^ | 1820 | 3627 | 8825 | 41.1 [39.8-42.5] | | 970 | 1880 | 5083 | 37.0 [35.3-38.7] | | 643 | 1303 | 2962 | 44.0 [41.7-46.4] | | 131 | 285 | 487 | 58.5 [52.1-65.7] | | 76 | 159 | 293 | 54.2 [46.4-63.3] | |
| Anal chlamydia | 1550 | 2882 | 8825 | 32.7 [31.5-33.9] | | 814 | 1468 | 5083 | 28.9 [27.4-30.4] | | 555 | 1045 | 2962 | 35.3 [33.2-37.5] | | 117 | 244 | 487 | 50.1 [44.2-56.8] | | 64 | 125 | 293 | 42.6 [35.8-50.8] | |
| Urogenital chlamydia | 621 | 854 | 8825 | 9.7 [9.0-10.3] | | 343 | 470 | 5083 | 9.2 [8.4-10.1] | | 220 | 306 | 2962 | 10.3 [9.2-11.6] | | 42 | 58 | 487 | 11.9 [9.2-15.4] | | 16 | 20 | 293 | 6.8 [4.4-10.6] | |
| Oropharyngeal chlamydia | 424 | 494 | 8825 | 5.6 [5.1-6.1] | | 206 | 236 | 5083 | 4.6 [4.1-5.3] | | 160 | 189 | 2962 | 6.4 [5.5-7.4] | | 34 | 38 | 487 | 7.8 [5.7-10.7] | | 24 | 31 | 293 | 10.6 [7.4-15.0] | |
| LGV | 269 | 326 | 8825 | 3.7 [3.3-4.1] | | 149 | 188 | 5083 | 3.7 [3.2-4.3] | | 99 | 114 | 2962 | 3.8 [3.2-4.6] | | 18 | 21 | 487 | 4.3 [2.8-6.6] | | 3 | 3 | 293 | 1.0 [0.3-3.2] | |
| **Gonorrhoea** |  |  |  |  |  |  |  |  |  |  |  |  |  |  |  |  |  |  |  |  |  |  |  |  |  |
| Any gonorrhoea^c^ | 2104 | 4679 | 8825 | 53.0 [51.5-54.6] | | 1105 | 2373 | 5083 | 46.7 [44.8-48.6] | | 762 | 1768 | 2962 | 59.7 [57.0-62.5] | | 141 | 338 | 487 | 69.4 [62.4-77.2] | | 96 | 200 | 293 | 68.2 [59.3-78.3] | |
| Anal gonorrhoea | 1580 | 3171 | 8825 | 35.9 [34.7-37.2] | | 817 | 1580 | 5083 | 31.1 [29.6-32.7] | | 570 | 1215 | 2962 | 41.0 [38.8-43.4] | | 115 | 242 | 487 | 49.7 [43.8-56.4] | | 78 | 134 | 293 | 45.7 [38.6-54.1] | |
| Urogenital gonorrhoea | 505 | 753 | 8825 | 8.5 [7.9-9.2] | | 258 | 359 | 5083 | 7.1 [6.4-7.8] | | 182 | 300 | 2962 | 10.1 [9.0-11.3] | | 46 | 67 | 487 | 13.8 [10.8-17.5] | | 19 | 27 | 293 | 9.2 [6.3-13.4] | |
| Oropharyngeal gonorrhoea | 1652 | 2825 | 8825 | 32.0 [30.9-33.2] | | 840 | 1387 | 5083 | 27.3 [25.9-28.8] | | 616 | 1094 | 2962 | 36.9 [34.8-39.2] | | 122 | 209 | 487 | 42.9 [37.5-49.1] | | 74 | 135 | 293 | 46.0 [38.9-54.5] | |
| **Infectious syphilis**^d^ | 560 | 661 | 8825 | 7.5 [6.9-8.1] | | 312 | 367 | 5083 | 7.2 [6.5-8.0] | | 188 | 227 | 2962 | 7.7 [6.7-8.7] | | 42 | 48 | 487 | 9.9 [7.4-13.1] | | 18 | 19 | 293 | 6.5 [4.1-10.2] | |

**Abbreviations:** CI, confidence interval; IR, incidence rate; No., number; PrEP, pre-exposure prophylaxis; PY, person-years; STI, sexually transmitted infection;
^a^ We defined any bacterial STI as chlamydia, gonorrhoea or infectious syphilis. Concurrent infections of different bacterial STIs were counted separately, while those of the same bacterium at different anatomical locations were counted as a single infection.
^b^ We defined any anal STI as any anorectal chlamydia or anorectal gonorrhoea, whereby concurrent infections of anorectal chlamydia and gonorrhoea were counted separately.
^c^Based on urogenital, anorectal, and oropharyngeal samples
^d^Syphilis stage 1, stage 2, or recent latent infection

**Supplementary Table 2.** Incidence of sexually transmitted infections while on PrEP, among individuals from prioritized subpopulations with ≥1 PrEP follow-up visit in the national PrEP program in Amsterdam, the Netherlands (1 July 2019 and 1 February 2024).

|  | **Younger than 25 (n=873)** | | | | | **Transgender or gender diverse (n=206)** | | | | | **Sex worker (n=325)** | | | | | **Uninsured (n=186)** | | | | | **Born in LMIC (n=1,218)** | | | | |
| --- | --- | --- | --- | --- | --- | --- | --- | --- | --- | --- | --- | --- | --- | --- | --- | --- | --- | --- | --- | --- | --- | --- | --- | --- | --- |
|  | No.  people  with an  STI | No. STIs | PY | IR (events/100PY) (95% CI) | | No. people with an STI | No. STIs | PY | IR (events/100PY) (95% CI) | | No.  people  with an  STI | No. STIs | PY | IR (events/100PY) (95% CI) | | No.  people  with an  STI | No. STIs | PY | IR (events/100PY) (95% CI) | | No.  people  with an  STI | No. STIs | PY | IR (events/100PY) (95% CI) | |
| **Any STI**^a^ | 547 | 1752 | 1453 | 120.6 [114.9-126.7] | | 108 | 250 | 289 | 86.5 [76.4-98.2] | | 208 | 700 | 509 | 137.4 [127.3-148.5] | | 122 | 398 | 283 | 140.7 [127.4-155.7] | | 761 | 2843 | 2398 | 118.5 [114.0-123.3] | |
| **Anal STI**^b^ | 456 | 1305 | 1453 | 89.8 [82.1-98.6] | | 82 | 160 | 289 | 55.3 [42.9-72.7] | | 169 | 486 | 509 | 95.4 [83.2-110.1] | | 100 | 271 | 283 | 95.8 [81.2-113.9] | | 607 | 1905 | 2398 | 79.4 [73.1-86.5] | |
| **Chlamydia** |  |  |  |  |  |  |  |  |  |  |  |  |  |  |  |  |  |  |  |  |  |  |  |  |  |
| Any chlamydia^c^ | 356 | 685 | 1453 | 47.2 [43.7-50.8] | | 64 | 112 | 289 | 38.7 [32.2-46.6] | | 134 | 290 | 509 | 56.9 [50.8-63.9] | | 81 | 159 | 283 | 56.2 [48.1-65.7] | | 533 | 1173 | 2398 | 48.9 [46.2-51.8] | |
| Anal chlamydia | 324 | 589 | 1453 | 40.5 [37.4-44.0] | | 53 | 90 | 289 | 31.1 [25.3-38.3] | | 112 | 228 | 509 | 44.8 [39.3-51.0] | | 68 | 123 | 283 | 43.5 [36.4-51.9] | | 454 | 933 | 2398 | 38.9 [36.5-41.5] | |
| Urogenital chlamydia | 102 | 133 | 1453 | 9.2 [7.7-10.9] | | 13 | 15 | 289 | 5.2 [3.1-8.6] | | 38 | 52 | 509 | 10.2 [7.8-13.4] | | 25 | 31 | 283 | 11.0 [7.7-15.6] | | 177 | 255 | 2398 | 10.6 [9.4-12.0] | |
| Oropharyngeal chlamydia | 91 | 106 | 1453 | 7.3 [6.0-8.8] | | 20 | 26 | 289 | 9.0 [6.1-13.2] | | 39 | 48 | 509 | 9.4 [7.1-12.5] | | 20 | 23 | 283 | 8.1 [5.4-12.2] | | 145 | 175 | 2398 | 7.3 [6.3-8.5] | |
| LGV | 42 | 49 | 1453 | 3.4 [2.5-4.5] | | 1 | 1 | 289 | 0.3 [0.0-2.5] | | 12 | 12 | 509 | 2.4 [1.3-4.1] | | 6 | 6 | 283 | 2.1 [1.0-4.7] | | 85 | 99 | 2398 | 4.1 [3.4-5.0] | |
| **Gonorrhoea** |  |  |  |  |  |  |  |  |  |  |  |  |  |  |  |  |  |  |  |  |  |  |  |  |  |
| Any gonorrhoea^c^ | 433 | 964 | 1453 | 66.4 [62.3-70.7] | | 73 | 121 | 289 | 41.8 [35.0-50.0] | | 166 | 368 | 509 | 72.3 [65.2-80.0] | | 100 | 217 | 283 | 76.7 [67.2-87.6] | | 603 | 1466 | 2398 | 61.1 [58.1-64.3] | |
| Anal gonorrhoea | 346 | 716 | 1453 | 49.3 [45.8-53.0] | | 49 | 70 | 289 | 24.2 [19.2-30.6] | | 137 | 258 | 509 | 50.7 [44.8-57.2] | | 81 | 148 | 283 | 52.3 [44.5-61.5] | | 454 | 972 | 2398 | 40.5 [38.1-43.2] | |
| Urogenital gonorrhoea | 97 | 126 | 1453 | 8.7 [7.3-10.3] | | 13 | 15 | 289 | 5.2 [3.1-8.6] | | 43 | 62 | 509 | 12.2 [9.5-15.6] | | 24 | 36 | 283 | 12.7 [9.2-17.6] | | 162 | 287 | 2398 | 12.0 [10.7-13.4] | |
| Oropharyngeal gonorrhoea | 370 | 636 | 1453 | 43.8 [40.5-47.3] | | 57 | 84 | 289 | 29.1 [23.5-36.0] | | 131 | 230 | 509 | 45.2 [39.7-51.4] | | 82 | 141 | 283 | 49.9 [42.3-58.8] | | 474 | 885 | 2398 | 36.9 [34.5-39.4] | |
| **Infectious syphilis**^d^ | 90 | 103 | 1453 | 7.1 [5.8-8.6] | | 16 | 17 | 289 | 5.9 [3.7-9.5] | | 38 | 42 | 509 | 8.2 [6.1-11.2] | | 21 | 22 | 283 | 7.8 [5.1-11.8] | | 169 | 204 | 2398 | 8.5 [7.4-9.8] | |

**Abbreviations:** CI, confidence interval; IR, incidence rate; No., number; PrEP, pre-exposure prophylaxis; PY, person-years; STI, sexually transmitted infection;
^a^ We defined any bacterial STI as chlamydia, gonorrhoea or infectious syphilis. Concurrent infections of different bacterial STIs were counted separately, while those of the same bacterium at different anatomical locations were counted as a single infection.
^b^ We defined any anal STI as any anorectal chlamydia or anorectal gonorrhoea, whereby concurrent infections of anorectal chlamydia and gonorrhoea were counted separately.
^c^Based on urogenital, anorectal, and oropharyngeal samples
^d^Syphilis stage 1, stage 2, or recent latent infection

**Supplementary Table 3.** Comparison of the incidence of grouped and specific STIs between prioritized and non-prioritized populations enrolled in the national PrEP program in Amsterdam, the Netherlands (1 July 2019-1 February 2024).

|  | Priority  criterion met | | Priority criterion not met | | Crude | | | Adjusted^e^ | | |
| --- | --- | --- | --- | --- | --- | --- | --- | --- | --- | --- |
|  | IR/100 py (95% CI) | | IR/100 py (95% CI) | | IRR (95% CI) | | p-value^f^ | aIRR (95% CI) | | p-value^f^ |
| A) <25 years (versus >= 25) |  |  |  |  |  |  |  |  |  |  |
| Any STI^a^ | 120.6 | [114.9-126.7] | 97.9 | [95.5-100.3] | 1.23 | [1.17-1.30] | <0.0001 | 1.24 | [1.14-1.35] | <0.0001 |
| Anal STI^b^ | 89.8 | [84.9-95.1] | 64.4 | [62.5-66.4] | 1.39 | [1.31-1.48] | <0.0001 | 1.43 | [1.28-1.58] | <0.0001 |
| Any chlamydia^c^ | 47.2 | [43.7-50.8] | 39.9 | [38.5-41.4] | 1.18 | [1.09-1.28] | 0.00011 | 1.16 | [1.05-1.28] | 0.0050 |
| Any gonorrhoea^c^ | 66.4 | [62.3-70.7] | 50.4 | [48.8-52.0] | 1.32 | [1.23-1.41] | <0.0001 | 1.27 | [1.17-1.39] | <0.0001 |
| Infectious syphilis^d^ | 7.1 | [5.8-8.6] | 7.6 | [7.0-8.2] | 0.94 | [0.75-1.16] | 0.55 | 0.91 | [0.74-1.14] | 0.42 |
| B) Transgender or genderdiverse (versus cisgender) | | |  |  |  |  |  |  |  |  |
| Any STI^a^ | 86.5 | [76.4-98.2] | 102.1 | [99.9-104.4] | 0.85 | [0.74-0.96] | 0.0087 | 0.72 | [0.60-0.87] | 0.00052 |
| Anal STI^b^ | 55.3 | [47.5-64.9] | 69.0 | [67.2-70.9] | 0.80 | [0.68-0.94] | 0.0050 | 0.69 | [0.55-0.87] | 0.0019 |
| Any chlamydia^c^ | 38.7 | [32.2-46.6] | 41.2 | [39.8-42.6] | 0.94 | [0.77-1.14] | 0.53 | 0.67 | [0.52-0.86] | 0.0015 |
| Any gonorrhoea^c^ | 41.8 | [35.0-50.0] | 53.4 | [51.9-55.0] | 0.78 | [0.65-0.94] | 0.0062 | 0.56 | [0.45-0.71] | <0.0001 |
| Infectious syphilis^d^ | 5.9 | [3.7-9.5] | 7.5 | [7.0-8.1] | 0.78 | [0.45-1.26] | 0.31 | 0.67 | [0.39-1.15] | 0.15 |
| C) Sex worker (versus no sex worker) |  |  |  |  |  |  |  |  |  |  |
| Any STI^a^ | 137.4 | [127.3-148.5] | 99.4 | [97.2-101.7] | 1.38 | [1.28-1.49] | <0.0001 | 1.41 | [1.22-1.63] | <0.0001 |
| Anal STI^b^ | 95.4 | [97.1-104.7] | 66.9 | [65.1-68.8] | 1.42 | [1.30-1.56] | <0.0001 | 1.42 | [1.19-1.69] | 0.00012 |
| Any chlamydia^c^ | 56.9 | [50.8-63.9] | 40.1 | [38.8-41.5] | 1.42 | [1.25-1.60] | <0.0001 | 1.41 | [1.18-1.70] | 0.00023 |
| Any gonorrhoea^c^ | 72.3 | [65.2-80.0] | 51.8 | [50.3-53.4] | 1.39 | [1.25-1.55] | <0.0001 | 1.42 | [1.20-1.67] | <0.0001 |
| Infectious syphilis^d^ | 8.2 | [6.1-11.2] | 7.4 | [6.9-8.1] | 1.11 | [0.79-1.52] | 0.51 | 1.21 | [0.82-1.77] | 0.33 |
| D) Without health insurance (versus insured) |  |  |  |  |  |  |  |  |  |  |
| Any STI^a^ | 140.7 | [127.4-155.7] | 100.3 | [98.1-102.6] | 1.40 | [1.27-1.55] | <0.0001 | 1.29 | [1.08-1.53] | 0.0034 |
| Anal STI^b^ | 95.8 | [84.8-108.6] | 67.7 | [65.9-69.6] | 1.42 | [1.25-1.60] | <0.0001 | 1.36 | [1.11-1.67] | 0.0032 |
| Any chlamydia^c^ | 56.2 | [48.1-65.7] | 40.6 | [39.3-42.0] | 1.38 | [1.17-1.62] | 0.00013 | 1.19 | [0.96-1.49] | 0.11 |
| Any gonorrhoea^c^ | 76.7 | [67.2-87.6] | 52.2 | [50.7-53.8] | 1.47 | [1.28-1.68] | <0.0001 | 1.31 | [1.08-1.58] | 0.0064 |
| Infectious syphilis^d^ | 7.8 | [5.1-11.8] | 7.5 | [6.9-8.1] | 1.04 | [0.65-1.59] | 0.83 | 0.99 | [0.61-1.59] | 0.95 |
| E) Migrant from an LMIC (versus born in high income country) | | | | | | | | | | |
| Any STI^a^ | 118.5 | [114.0-123.3] | 95.3 | [92.8-97.8] | 1.24 | [1.19-1.30] | <0.0001 | 1.16 | [1.08-1.26] | <0.0001 |
| Anal STI^b^ | 79.4 | [75.7-83.3] | 64.5 | [62.5-66.6] | 1.23 | [1.17-1.30] | <0.0001 | 1.16 | [1.05-1.28] | 0.0039 |
| Any chlamydia^c^ | 48.9 | [46.2-51.8] | 38.2 | [36.7-39.7] | 1.28 | [1.19-1.37] | <0.0001 | 1.21 | [1.11-1.33] | <0.0001 |
| Any gonorrhoea^c^ | 61.1 | [58.1-64.3] | 50.0 | [48.3-51.8] | 1.22 | [1.15-1.30] | <0.0001 | 1.15 | [1.06-1.25] | 0.00054 |
| Infectious syphilis^d^ | 8.5 | [7.4-9.8] | 7.1 | [6.5-7.8] | 1.20 | [1.01-1.41] | 0.035 | 1.18 | [0.99-1.40] | 0.073 |

Abbreviations: a(IRR), (adjusted) incidence rate ratio; CI, confidence interval; IR, incidence rate; No., number; PrEP, pre-exposure prophylaxis; PY, person-years; STI, sexually transmitted infection;
^a^ We defined any bacterial STI as chlamydia, gonorrhoea or infectious syphilis. Concurrent infections of different bacterial STIs were counted separately, while those of the same bacterium at different anatomical locations were counted as a single infection.
^b^ We defined any anal STI as any anorectal chlamydia or anorectal gonorrhoea, whereby concurrent infections of anorectal chlamydia and gonorrhoea were counted separately.
^c^Based on urogenital, anorectal, and oropharyngeal samples
^d^Syphilis stage 1, stage 2, or recent latent infection
^e^Adjusted for other priority groups if applicable (i.e., variables in panel A-E but not F-H), calendar time, and individual yearly STI testing frequency. Calendar time and STI testing frequency were modelled as cubic splines with four knots at the 5^th^, 35^th^, 65^th^ and 95^th^ percentile..

^f^p-values were calculated using the Wald χ^2^ test

**Supplementary Table 4.** Comparison of the incidence of grouped and specific STIs by number of priority criteria, among individuals enrolled in the national PrEP program in Amsterdam, the Netherlands (1 July 2019-1 February 2024).

|  | IR/100 py (95% CI) | | Crude IRR (95% CI) | | p-value^f^ | aIRR^e^ (95% CI) | | p-value^f^ |
| --- | --- | --- | --- | --- | --- | --- | --- | --- |
| A) No priority criteria met |  |  |  |  |  |  |  |  |
| Any STI^a^ | 90.9 | [88.2-93.7] | Ref. |  |  | Ref. |  |  |
| Anal STI^b^ | 60.0 | [57.8-62.3] | Ref. |  |  | Ref. |  |  |
| Any chlamydia^c^ | 37.0 | [35.3-38.7] | Ref. |  |  | Ref. |  |  |
| Any gonorrhoea^c^ | 46.7 | [44.8-48.6] | Ref. |  |  | Ref. |  |  |
| Infectious syphilis^d^ | 7.2 | [6.5-8.0] | Ref. |  |  | Ref. |  |  |
| B) One priority criterion met |  |  |  |  |  |  |  |  |
| Any STI^a^ | 111.4 | [107.5-115.4] | 1.23 | [1.17-1.28] | <0.0001 | 1.17 | [1.09-1.26] | <0.0001 |
| Anal STI^b^ | 76.3 | [73.0-79.7] | 1.27 | [1.20-1.34] | <0.0001 | 1.21 | [1.10-1.33] | <0.0001 |
| Any chlamydia^c^ | 44.0 | [41.7-46.4] | 1.19 | [1.11-1.28] | <0.0001 | 1.14 | [1.05-1.24] | 0.0030 |
| Any gonorrhoea^c^ | 59.7 | [57.0-62.5] | 1.28 | [1.20-1.36] | <0.0001 | 1.22 | [1.13-1.32] | <0.0001 |
| Infectious syphilis^d^ | 7.7 | [6.7-8.7] | 1.06 | [0.90-1.26] | 0.48 | 1.04 | [0.87-1.24] | 0.66 |
| C) Two priority criteria met |  |  |  |  |  |  |  |  |
| Any STI^a^ | 137.8 | [127.3-149.3] | 1.52 | [1.40-1.64 | <0.0001 | 1.44 | [1.27-1.65] | <0.0001 |
| Anal STI^b^ | 99.8 | [91.0-109.7] | 1.66 | [1.51-1.83] | <0.0001 | 1.60 | [1.36-1.89] | <0.0001 |
| Any chlamydia^c^ | 58.5 | [52.1-65.7] | 1.58 | [1.39-1.79] | <0.0001 | 1.51 | [1.29-1.76] | <0.0001 |
| Any gonorrhoea^c^ | 69.4 | [62.4-77.2] | 1.49 | [1.32-1.67] | <0.0001 | 1.42 | [1.23-1.63] | <0.0001 |
| Infectious syphilis^d^ | 9.9 | [7.4-13.1] | 1.37 | [0.99-1.85] | 0.049 | 1.31 | [0.87-1.75] | 0.075 |
| D) Three or more priority criteria met | | | | | | | | |
| Any STI^a^ | 128.8 | [116.3-143.1] | 1.42 | [1.27-1.57] | <0.0001 | 1.71 | [1.47-1.98] | <0.0001 |
| Anal STI^b^ | 88.3 | [78.0-100.3] | 1.47 | [1.29-1.67] | <0.0001 | 1.85 | [1.54-2.23] | <0.0001 |
| Any chlamydia^c^ | 54.2 | [46.4-63.3] | 1.47 | [1.24-1.72] | <0.0001 | 1.70 | [1.39-2.07] | <0.0001 |
| Any gonorrhoea^c^ | 68.2 | [59.3-78.3] | 1.46 | [1.26-1.69] | <0.0001 | 1.76 | [1.49-2.07] | <0.0001 |
| Infectious syphilis^d^ | 6.5 | [4.1-10.2] | 0.90 | [0.53-1.42] | 0.67 | 1.03 | [0.66-1.61] | 0.71 |

Abbreviations: a(IRR), (adjusted) incidence rate ratio; CI, confidence interval; IR, incidence rate; PrEP, pre-exposure prophylaxis; PY, person-years; STI, sexually transmitted infection;

^a^We defined any bacterial STI as chlamydia, gonorrhoea or infectious syphilis. Concurrent infections of different bacterial STIs were counted separately, while those of the same bacterium at different anatomical locations were counted as a single infection.
^b^ We defined any anal STI as any anorectal chlamydia or anorectal gonorrhoea, whereby concurrent infections of anorectal chlamydia and gonorrhoea were counted separately.
^c^Based on urogenital, anorectal, and oropharyngeal samples
^d^Syphilis stage 1, stage 2, or recent latent infection
^e^Adjusted for other priority groups if applicable (i.e., variables in panel A-E but not F-H), calendar time, and individual yearly STI testing frequency. Calendar time and STI testing frequency were modelled as cubic splines with four knots at the 5^th^, 35^th^, 65^th^ and 95^th^ percentile.

^f^p-values were calculated using the Wald χ^2^ test

**Supplementary Table 5.** Odds ratios for early LTFU among 4,176 individuals who enrolled in the national PrEP program in Amsterdam, the Netherlands between 1 July 2019 and 1 February 2023.

|  |  | |  | | Univariable | |  | Multivariable, model 1^b^ | |  | Multivariable, model 2^c^ | |
| --- | --- | --- | --- | --- | --- | --- | --- | --- | --- | --- | --- | --- |
|  | n/N^a^ | (%) | n/N^a^ | (%) | OR [95% CI] | p-value |  | aOR [95% CI] | p-value |  | aOR [95% CI] | p-value |
| **A) Specific priority criteria** | Priority criterion met | | Priority criterion  not met | |  |  |  |  |  |  |  |  |
| Age <25 years (*vs* ≥25 years) | 174/927 | (18.8%) | 320/3,249 | (9.9%) | 2.12 [1.73-2.59] | <0.0001 |  | 2.12 [1.72-2.60] | <0.0001 |  | 2.03 [1.63-2.53] | <0.0001 |
| TGD (*vs* cisgender) | 70/238 | (29.4%) | 424/3,939 | (10.8%) | 3.45 [2.57-4.64] | <0.0001 |  | 1.50 [1.04 -2.15] | 0.029 |  | 1.37 [0.91-2.07] | 0.13 |
| Sex worker (*vs* no sex worker) | 126/405 | (31.1%) | 368/3,771 | (9.8%) | 4.18 [3.30-5.30] | <0.0001 |  | 3.13 [2.31-4.25] | <0.0001 |  | 3.46 [2.31-5.19] | <0.0001 |
| No health insurance (*vs* insured) | 49/201 | (24.4%) | 445/3,975 | (11.2%) | 2.56 [1.83-3.58] | <0.0001 |  | 0.93 [0.62-1.39] | 0.72 |  | 0.88 [0.56-1.38] | 0.58 |
| Born in LMIC (*vs* born in HIC) | 199/1,235 | (16.1%) | 295/2,941 | (10.0%) | 1.72 [1.42-2.09] | <0.0001 |  | 1.36 [1.09-1.69] | 0.0062 |  | 1.29 [1.02-1.63] | 0.037 |
| **B) Number of priority criteria** | Number of priority criteria met | |  |  |  |  |  |  |  |  |  |  |
| 0 | 155/2,099 | (7.4%) |  |  | Ref. |  |  | n/a |  |  | Ref. |  |
| 1 | 173/1,497 | (11.6%) |  |  | 1.64 [1.31-2.06] | <0.0001 |  | n/a |  |  | 1.61 [1.27-2.04] | <0.0001 |
| 2 | 91/337 | (27.0%) |  |  | 4.64 [3.47-6.21] | <0.0001 |  | n/a |  |  | 4.04 [2.94-5.57] | <0.0001 |
| ≥3 | 75/243 | (30.9%) |  |  | 5.60 [4.08-7.69] | <0.0001 |  | n/a |  |  | 3.81 [2.40-6.04] | <0.0001 |

Abbreviations, (a)OR, (adjusted) odds ratio; CI, confidence interval; HIC, high-income country; LMIC, low- or middle-income country; LTFU, loss-to-follow-up; n, number; n/a, not applicable; PrEP, pre-exposure prophylaxis; TGD, transgender or gender-diverse; vs, versus;
^a^The denominator included all individuals who enrolled before 1 February 2023, and who therefore had to opportunity to be considered ‘early LTFU’ before administrative censoring on 1 February 2024.
^b^We adjusted ORs for the other higher-priority criteria if applicable (i.e., in A but not in B).

^c^We adjusted ORs for the other higher-priority criteria if applicable (i.e., in A but not in B), number of sex partners, any condomless anal sex with a casual partner, and any chemsex, all in the past six months. Number of partners was modelled as cubic splines with four knots at the 5^th^, 35^th^, 65^th^ and 95^th^ percentile.

**Supplementary Table 6.** Hazard ratios for later LTFU among 4,230 individuals with ≥1 PrEP follow-up visit before 1 February 2023, in the national PrEP program in Amsterdam, the Netherlands (1 July 2019-1 February 2024).

|  |  | | |  |  | | |  | Univariable | |  | Multivariable, model 1^a^ | |  | Multivariable, model 2^b^ | |
| --- | --- | --- | --- | --- | --- | --- | --- | --- | --- | --- | --- | --- | --- | --- | --- | --- |
|  | N. LTFU | PY | IR  (LTFU/100 PY) |  | N. LTFU | PY | IR  (LTFU/100 PY) |  | HR [95%CI] | p-value |  | aHR [95%CI] | p-value^c^ |  | aHR [95%CI] | p-value^c^ |
| **A) Specific priority criteria** | Priority criterion met | | |  | Priority criterion not met | | |  |  |  |  |  |  |  |  |  |
| <25 years (*vs* ≥25 years) | 256 | 1596 | 16.0 [14.2-18.1] |  | 667 | 7762 | 8.7 [8.1-9.4] |  | 1.89 [1.63-2.18] | <0.0001 |  | 1.71 [1.48-1.98] | <0.0001 |  | 1.69 [1.45-1.97] | <0.0001 |
| TGD (*vs* cisgender) | 76 | 338 | 22.5 [18.0-28.1] |  | 857 | 9021 | 9.5 [8.9-10.2] |  | 2.75 [2.16-3.49] | <0.0001 |  | 1.56 [1.16-2.11] | 0.0038 |  | 1.74 [1.27-2.39] | 0.00056 |
| Sex work (*vs* no sex work) | 133 | 593 | 22.4 [18.9-26.6] |  | 800 | 8766 | 9.1 [8.5-9.8] |  | 2.69 [2.25-3.22] | <0.0001 |  | 2.12 [1.64-2.74] | <0.0001 |  | 3.03 [2.31-3.98] | <0.0001 |
| Uninsured (*vs* insured) | 54 | 306 | 17.6 [13.5-23.0] |  | 879 | 9052 | 9.7 [9.1-10.4] |  | 1.93 [1.47-2.53] | <0.0001 |  | 1.00 [0.73-1.38] | 0.99 |  | 1.10 [0.80-1.52] | 0.55 |
| Born in LMIC (*vs* born in HIC) | 273 | 2525 | 10.8 [9.6-12.2] |  | 660 | 6834 | 9.7 [8.9-10.4] |  | 1.15 [1.00-1.33] | 0.048 |  | 1.00 [0.83-1.14] | 0.74 |  | 0.93 [0.79-1.10] | 0.42 |
| **B) Number of priority criteria** | Number of priority criteria met | | |  |  |  |  |  |  |  |  |  |  |  |  |  |
| 0 | 408 | 5345 | 7.6 [6.9-8.4] |  |  |  |  |  | Ref. |  |  | Ref. |  |  | Ref. |  |
| 1 | 354 | 3134 | 11.3 [10.2-12.5] |  |  |  |  |  | 1.52 [1.32-1.76] | <0.0001 |  | 1.47 [1.27-1.69] | <0.0001 |  | 1.40 [1.20-1.64] | <0.0001 |
| 2 | 102 | 545 | 18.7 [15.4-22.7] |  |  |  |  |  | 2.58 [2.07-3.20] | <0.0001 |  | 2.50 [2.01-3.11] | <0.0001 |  | 2.40 [1.91-3.02] | <0.0001 |
| ≥3 | 69 | 336 | 20.6 [16.2-26.0] |  |  |  |  |  | 3.10 [2.42-3.99] | <0.0001 |  | 2.84 [2.21-3.64] | <0.0001 |  | 3.72 [2.71-5.12] | <0.0001 |

**Abbreviations:** (a)HR, (adjusted) hazards ratio; CI, confidence interval; HIC, high-income country; IR, incidence rate; LMIC, low- or middle-income country; n, number; n/a, not applicable; PrEP, pre-exposure prophylaxis; PY, person-years; TGD, transgender or gender-diverse; vs: versus;
NB: we assumed the timing of LTFU as 12 months after the last recorded PrEP visit.

Missing data: number of sex partners (n=35) , any any condomless anal sex with a casual partner (n=101), any chemsex (n=28)
^a^Model 1: adjusted for the other priority criteria (only in A) and enrolment date. Enrolment date was modelled as cubic splines with four knots at the 5^th^, 35^th^, 65^th^ and 95^th^ percentile.
^b^Model 2: adjusted for the other priority criteria (only in A), enrolment date, and the following sexual behavior variables (all referring to the six months preceding one’s last PrEP visit): number of sex partners, any condomless anal sex with a casual partner, and any chemsex. Enrolment date and number of sex partners were modelled as cubic splines with four knots at the 5^th^, 35^th^, 65^th^ and 95^th^ percentile.

^c^p-values were calculated using the Wald χ^2^ test

**Supplementary Table 7.** Incidence rate ratios for repeat enrolment among 1,656 individuals who previously exited the national PrEP program in Amsterdam, the Netherlands (1 July 2019-1 February 2024)

|  |  | | | |  |  | | | |  | Univariable | |  | Multivariable^a^ | |
| --- | --- | --- | --- | --- | --- | --- | --- | --- | --- | --- | --- | --- | --- | --- | --- |
|  | n/N | (%) | PY | IR per year (re-enrolments / PY) |  | n/N | (%) | PY | IR per year (re-enrolments / PY) |  | IRR (95% CI) | p-value^b^ |  | aIRR (95% CI) | p-value^b^ |
| **A)  Specific priority criterion** | Priority criterion met | | | |  | Priority criterion not met | | | |  |  |  |  |  |  |
| Age <25 years (*vs* ≥25 years) | 109/499 | (21.8%) | 615 | 0.18 [0.15-0.21] |  | 272/1,175 | (23.5%) | 1450 | 0.19 [0.17-0.21] |  | 0.95 [0.77-1.18] | 0.62 |  | 0.94 [0.75-1.18] | 0.60 |
| TGD (*vs* cisgender) | 33/155 | (21.3%) | 171 | 0.19 [0.14-0.27] |  | 348/1,501 | (23.2%) | 1894 | 0.18 [0.17-0.20] |  | 1.05 [0.73-1.50] | 0.79 |  | 0.76 [0.50-1.13] | 0.18 |
| Sex worker (*vs* no sex worker) | 72/287 | (25.1%) | 325 | 0.22 [0.18-0.28] |  | 309/1,369 | (22.6%) | 1740 | 0.18 [0.16-0.20] |  | 1.25 [0.97-1.61] | 0.090 |  | 1.11 [0.82-1.51] | 0.49 |
| No health insurance (*vs* insured) | 30/116 | (25.9%) | 92 | 0.33 [0.23-0.47] |  | 351/1,540 | (22.8%) | 1973 | 0.18 [0.16-0.20] |  | 1.83 [1.26-2.66] | 0.0014 |  | 1.02 [0.68-1.53] | 0.93 |
| Born in LMIC (*vs* born in HIC) | 144/537 | (26.8%) | 625 | 0.23 [0.20-0.27] |  | 237/1,119 | (21.2%) | 1440 | 0.16 [0.14-0.19] |  | 1.40 [1.14-1.72] | 0.0014 |  | 1.29 [1.03-1.61] | 0.025 |
| **B)  Number of priority criteria** | Number of priority criteria met | | | |  |  |  |  |  |  |  |  |  |  |  |
| 0 | 143/668 | (21.4%) | 875 | 0.20 [0.17-0.23] |  |  |  |  |  |  | Ref. |  |  | n/a |  |
| 1 | 149/610 | (24.4%) | 763 | 0.16 [0.14-0.19] |  |  |  |  |  |  | 1.20 [0.95-1.50] | 0.13 |  | n/a |  |
| 2 | 49/219 | (22.4%) | 269 | 0.18 [0.14-0.24] |  |  |  |  |  |  | 1.11 [0.80-1.54] | 0.52 |  | n/a |  |
| ≥3 | 40/159 | (25.2%) | 157 | 0.25 [0.19-0.35] |  |  |  |  |  |  | 1.55 [1.09-2.21] | 0.014 |  | n/a |  |

Abbreviations, (a)IRR, (adjusted) incidence rate ratio; CI, confidence interval; HIC, high-income country; IR, incidence rate; LMIC, low- or middle-income country; n, number; n/a, not applicable; PrEP, pre-exposure prophylaxis; TGD, transgender or gender-diverse; vs, versus;

^a^IRRs were adjusted for the other priority criteria (only in A)
^b^p-values were calculated using the Wald χ^2^ test
